# Lithium Difluorophosphate and Fluoroethylene Carbonate Containing Electrolytes: How Instrumental Analytics Helps to Understand Synergistic Effects on Performance Improvement in High‐Voltage Li Battery Applications

**DOI:** 10.1002/smtd.70857

**Published:** 2026-07-27

**Authors:** Nick Fehlings, Alexandros Tsoufios, Tim Messink, Martin Winter, Simon Wiemers‐Meyer, Sascha Nowak

**Affiliations:** ^1^ MEET Battery Research Center University of Münster Münster Germany; ^2^ Helmholtz‐Institute Münster, IMD‐4 FZ Jülich Münster Germany

**Keywords:** analytical chemistry, DFP, electrolyte additive, electrolyte degradation, FEC, high voltage, HPLC, ICP‐OES, lithium ion battery, reverse‐engineering, transition metal dissolution

## Abstract

Sequential improvement of lithium‐ion batteries is an ongoing process that addresses operational factors such as high voltage, safety, and/or lifetime. Tailored electrolytes serve a critical role, as they are heavily involved in the formation of the solid electrolyte interphase (SEI) and the cathode electrolyte interphase (CEI). For high‐voltage applications, a properly formed CEI is essential to prevent continuous decomposition of active material or electrolyte. Preventing transition metal deposition on the negative electrode is important, as this may trigger the rollover effect, which results in a drastic loss of capacity. Specific additives employed in the electrolyte can help prevent these side reactions. This study investigates different electrolyte formulations combining various concentration ratios of fluoroethylene carbonate (FEC) and lithium difluorophosphate (DFP). Long‐term cycling at increased upper cut‐off voltages revealed that not only can the rollover effect occurring for FEC and EC as cosolvents be prevented, but also increased capacity retention by the addition of DFP. Transition metal deposition is drastically reduced for the employment of both DFP and FEC as cosolvents, revealing synergistic effects. Additionally, degradation products of in situ formed VC and DFP, which counteract the negative effect of FEC and EC, are revealed via instrumental analytics.

## Graphical abstract

In this study, electrolyte investigations in cells for high‐voltage applications are carried out. The electrolytes contain varying contents of FEC and EC, including the replacement of EC by FEC and the addition of the electrolyte additive DFP.

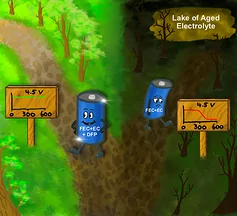

## 1 Introduction

The demand for higher energy density is essential for advancing the development of state‐of‐the‐art lithium‐ion batteries (LIB) [1]. However, high‐voltage applications (≥ 4.5 V) of LIBs complicate the utilization of commonly employed electrolytes, as the electrochemical decomposition is accelerated at elevated upper cutoff voltages (UCV). Ethylene carbonate is prone to undergoing severe decomposition, resulting in severe degradation of the electrolyte and increasing capacity fading [2, 3]. The so‐called solid electrolyte interphase (SEI) is formed at the negative electrode surface during cell formation and can prevent continuous electrolyte degradation [4, 5]. Structural changes of the negative electrode during cycling alter the properties of the SEI and may lead to SEI cracking [6]. Fresh (SEI‐unprotected) electrode surfaces are then exposed to the electrolyte, which leads to further electrolyte decomposition [4]. For increased UCV, parasitic side reactions further deteriorate the SEI, ultimately decreasing cell performance. One main factor is the rollover effect, which describes the turning point from linear to accelerated capacity fading and finally the failure of the cell [7, 8, 9]. The rollover effect is typically triggered by a combination of progressive loss of active lithium, transition metal dissolution from the cathode, thickening of the SEI, and/or lithium plating, where each subsequent cycle amplifies previous damage [10].

Especially for high voltage, the cathode undergoes increased material degradation, which results in transition metal dissolution as a main contributing factor for the rollover effect [11, 12]. Electrolyte additives can be employed in the electrolyte to prevent these challenges [13, 14, 15]. Commonly used additives include fluoroethylene carbonate (FEC), vinyl carbonate (VC), and lithium salts such as lithium difluoro phosphate (LiDFP) [13, 16, 17]. FEC and VC, which decompose before EC during formation, are employed to modify the SEI [2]. LiDFP has been investigated as a high‐voltage additive, primarily attributed to its ability to complex dissolved transition metal ions and suppress their deposition on the negative electrode [18, 19]. Klein et al. showed that the addition of DFP to the electrolyte for high‐voltage cathodes prevents the rollover effect by complexing TM, hindering deposition on the negative electrode surface [20, 21]. Literature showed that if FEC is used in the electrolyte, a more flexible SEI is formed with increased LiF content compared to the fluorine‐free solvent EC [22, 23]. Despite advantages, FEC employed with EC as a co‐solvent is not suitable for high‐voltage applications [24].

The use of multiple additives proved to be effective in considerably enhancing electrochemical behavior [21, 25, 26]. Zheng et al. investigated that the presence of DFP and FEC within the electrolyte can improve the SEI in SiC‐based batteries [27]. Additionally, Li et al. investigated FEC‐based electrolytes with the addition of DFP, focusing on the SEI composition, but no description of interactions between DFP and FEC was provided [18]. This decomposition of DFP and FEC was not described in the literature, yet elucidating mechanisms occurring in battery cells allows the battery composition to be tailored in aspects of hindering or even to make use of reactions.

Analytical methodologies are widely used to elucidate aging phenomena emerging during cycling or other conditions. Electrolyte analysis is conducted mostly via chromatographic methodologies like gas chromatography (GC), ion chromatography (IC), or high‐performance liquid chromatography (HPLC) coupled to mass analyzers (MS) [28]. During cell operation, the molecules polymerize, which then can be analyzed by HPLC and finally verified with MS^2^ fragmentation experiments revealing the occurring degradation mechanism [29, 30]. For investigation of transition metal deposition on negative electrodes, methods like inductively coupled plasma (ICP) coupled to optical emission spectroscopy (OES) or total x‐ray fluorescence analysis are used for analysis [31]. With the help of ICP‐OES, the quantification of even low‐concentrated analytes of transition metal ions is possible [32].

This study examines the synergistic influence of FEC as a co‐solvent and DFP as an electrolyte additive in a high‐voltage NMC||graphite cell using analytical methods. Long‐term electrochemical investigations with two different UCVs were applied to investigate the influence of DFP and FEC on formed degradation products emerging in the electrolyte. The correlation of degradation product formation and electrochemical performance provides insights into degradation pathways of FEC and DFP and their impact on high‐voltage cell aging.

## 2 Experimental

### 2.1 Chemicals and Materials

Ethyl methyl carbonate (EMC; 99.9%), ethylene carbonate (EC; 99.9%), fluoroethylene carbonate (FEC; 99.9%), and LiPF_6_ (99.89%) were obtained from E‐Lyte (Muenster, Germany). Lithium difluorophosphate (DFP; American Elements; purity: ≥ 99.9%) was employed as an additive. Furthermore, acetonitrile (ACN, hypergrade, Merck KGaA, Darmstadt) and deionized water (Barnstead GenPure xCAD Plus, Thermo Scientific, Waltham, USA) were used as eluents and for sample dilution. Electrolytes were formulated by an in‐house robotic high‐throughput system (HTS) in a glove box (O_2_, H_2_O < 0.1 ppm). All chemicals were used as received. All electrolytes contained 1 mol L^−1^ LiPF_6_, EMC (70 wt. %), and EC or FEC in varying concentrations shown in Table 1, with half of the electrolytes having DFP added.

**TABLE 1:** Compositions of all electrolytes investigated for long‐term cycling.

STD	F10	F15	F20	F30
EC:EMC 3:7	EC:FEC:EMC 2:1:7	EC:FEC:EMC 1.5:1.5:7	EC:FEC:EMC 1:2:7	FEC:EMC 3:7
STD+DFP	F10+DFP	F15+DFP	F20+DFP	F30+DFP
EC:EMC 3:7 + 1wt% DFP	EC:FEC:EMC 2:1:7 + 1wt% DFP	EC:FEC:EMC 1.5:1.5:7 + 1wt% DFP	EC:FEC:EMC 1:2:7 + 1wt% DFP	FEC:EMC 3:7 + 1wt% DFP

### 2.2 Cell Assembly and Electrochemical Operation

Commercially available LIB pouch cells (Li‐Fun NMC622||graphite, 200 mAh) were dried under reduced pressure for 24 h at 80°C. The areal densities of the cathode and anode are 12.18 and 9.15 mg/cm^2^, respectively. The cells were filled with 750 µL of the respective electrolyte formulation (Table 1) and sealed with a relative pressure of −85 kPa for 5 s. After wetting for 6 h, three constant current (CC) charge (0.1 C) and CC discharge (0.1 C) cycles were performed in a voltage window between 4.2 and 3.0 V or 4.5 and 3.0 V at 20°C. After formation, long‐term cycling was performed for 600 CC (1.0 C) charge and 600 CC (1.0 C) discharge cycles at 20°C. For both UCVs, the same N/P ratio of 1.3 was used to exclude discrepancies in performance, and the applied pressure during cycling was 3.6 bar.

### 2.3 HPLC‐QToF Measurements

After electrochemical application, all cells were opened in a glovebox (O_2_, H_2_O < 0.1 ppm) and electrolytes were extracted via centrifugation of the electrode‐separator stack at 9500 rpm for 20 min. Investigation of electrochemically aged LIB electrolyte decomposition products was performed with a Nexera X2 UHPLC system (Shimadzu, Kyoto, Japan) hyphenated to a 6530 Q‐TOF (Agilent). Reversed‐phase chromatography was conducted on a ZORBAX SB‐C18 column (100 × 2.1 mm, 1.9 µm; Agilent Technologies, Inc., Santa Clara, CA, USA) at 40°C and a flow rate of 0.5 mL min^−1^. Sample preparation was conducted according to Henschel et al., while chromatographic separation was adapted [33]. The mobile phase consisted of 0.1 V% formic acid (A) and acetonitrile (B). The gradient started with 5% B from 0 to 1.9 min and increased to 70% B within 12.1 min. Subsequently, the mobile phase was kept constant at 60% B for 6 min. Finally, the column was equilibrated at 5% B for 4 min. The injection volume was 5 µL. To protect the QToF mass spectrometer from high concentrations of conducting salts, the flow line switched to the MS after 1.4 min. Ionization was performed in ESI(+) mode. The source parameters were gas temperature 350°C, drying gas flow 10 mL/min, nebulizer pressure 45 psi, VCap 3000 V. The mass range was set to a mass‐to‐charge (*m*/*z*) of 100–1700 and 90–1000 with an acquisition rate of 2 spectra/s. The collision‐induced energy was set to 12 eV. A mzmine software (version: 4.8.30, mzio) was used for LC‐MS data evaluation [34]. All samples were diluted 1:100 with ACN for measurements.

### 2.4 ICP‐OES Measurements

Transition metal deposition was investigated by microwave digestion‐assisted ICP‐OES measurements. Four 15 mm punchings per cell were dissolved in reverse *aqua regia* using a microwave system (Multiwave 7000, Anton Paar, Austria). The temperature was ramped linearly from 40°C to 260°C over a period of 12 min, then held at 260°C and a pressure of 110 bar for 30 min. ICP‐OES measurements were performed using an ARCOS (Spectro Analytical Instruments GmbH, Germany) equipped with a Scott‐style spray chamber, a cross‐flow nebulizer, and an axially positioned plasma torch. A coolant flow of 12.00 L/min, an auxiliary flow of 1.00 L/min, and a nebulizer flow of 0.9 L/min were applied with a plasma power of 1400 W. The following emission lines were observed: Co (228.616 nm), Mn (257.611 nm), and Ni (231.604 nm). The method and further parameters were adapted according to Vortmann‐Westhoven et al. and Evertz et al. [31, 35].

### 2.5 XPS Measurements

X‐ray photoelectron spectroscopy (XPS) measurements were performed on 12 mm samples punched from the center of the negative electrode sheet. A monochromatic Al Kα source (hν = 1486.6 eV) was used at an emission current of 10 mA with an accelerating voltage of 12 kV. The emission angle was 0, and the hemispherical analyzer was set to pass energy of 20 eV for electrode spectra. Core spectra were recorded in the following regions: F1s, C 1s, P 2p, and Li 1s. For sputter depth profiling, argon was used as an ion source, with a beam energy of 500 eV and an extractor current of 15 µA. Data evaluation was carried out with *CasaXPS* (v 2.3.23, Casa Software).

### 2.6 SEM Measurements

For scanning electron microscopy (SEM) analysis, electrode disks (12 mm) were punched out and analyzed with a SEM (Carl‐Zeiss CrossBeam 550, Carl Zeiss Microscopy GmbH) equipped with a field emission gun (Schottky‐type). The SEM images were recorded with an accelerating voltage of 3 kV.

## 3 Results and Discussion

### 3.1 Electrochemical Aging

10 different electrolytes were investigated in commercially available pouch cells (NMC622||AG) and cycled for 600 cycles (1.0 C charge and discharge). 5 of the electrolytes with two different UCV were already investigated by Fehlings et al., whereby the employment of FEC as a co‐solvent in concentrations of 0%, 10%, 15%, 20% and 30% (STD, F1, F15, F20, F30) was evaluated [24]. Increasing the FEC content revealed improved electrochemical performance for long‐term cycling with 4.2 V UCV (Figure 1a). F30 outperformed the other electrolytes due to no possible degradation reaction between FEC and EC and thus a combination of an improved SEI. Cycling at 4.5 V UCV showed counterintuitive findings, whereas the rollover effect for F10, F15 and F20 occurred after 200, 250 and 300 cycles, respectively (Figure 1c), again accelerated by the degradation reaction of FEC and EC‐related products. The corresponding capacity retention and charge–discharge curves for cycles 5, 50, 300 and 600 are shown in Figures S1–S5. The rollover effect can be described as the turning point where the linear capacity fading transitions into rapid capacity loss. Various factors such as progressive lithium loss, transition metal dissolution, lithium plating, and/or thickening of SEI are known to contribute to this [7, 8, 9]. Typically, after reaching the turning point of the rollover effect, each cycle then amplifies the capacity loss, resulting in accelerated cell failure. For both UCVs, the electrolyte F30, replacing EC with FEC, shows the best long‐term performance, matching previous data of Klein et al., which stated that EC has a worsening impact for high‐voltage LIBs [17]. For a 4.2 V UCV, the employment of DFP as an electrolyte additive increased the capacity for each electrolyte by up to 10 mAh (Figure 1b). Aside from the observed capacity improvement, the capacity trend for increased FEC content remains stable, indicating that FEC is compatible as a co‐solvent when DFP is included. All 10 electrolytes with 4.2 V UCV show no rollover effect, which is consistent with the literature that lower UCVs do not lead to this effect [36, 37]. The addition of DFP to electrolytes for 4.5 V UCV counteracts the rollover effect for every electrolyte (Figure 1d). Nevertheless, the trend toward higher capacity with increased FEC content remains the same, with F30 having the highest capacity. STD electrolytes, in contrast, show the worst capacity retention for every electrolyte. DFP in the electrolyte for higher UCVs results in increased concentrations of fluorophosphates, which then can prevent the rollover effect. The increased capacity of around 10 mAh after the addition of DFP can be attributed to less active loss during formation and the addition of Li, which counteracts active material loss. The worse capacity retention for FEC and EC being present (F10, F15, and F20) without DFP added can be completely negated, indicating the synergistic effect of FEC as a cosolvent with DFP as an additive. Even allowing the employment of lower FEC contents (F10–F15) for comparable capacity retention over 600 cycles.

**FIGURE 1:**
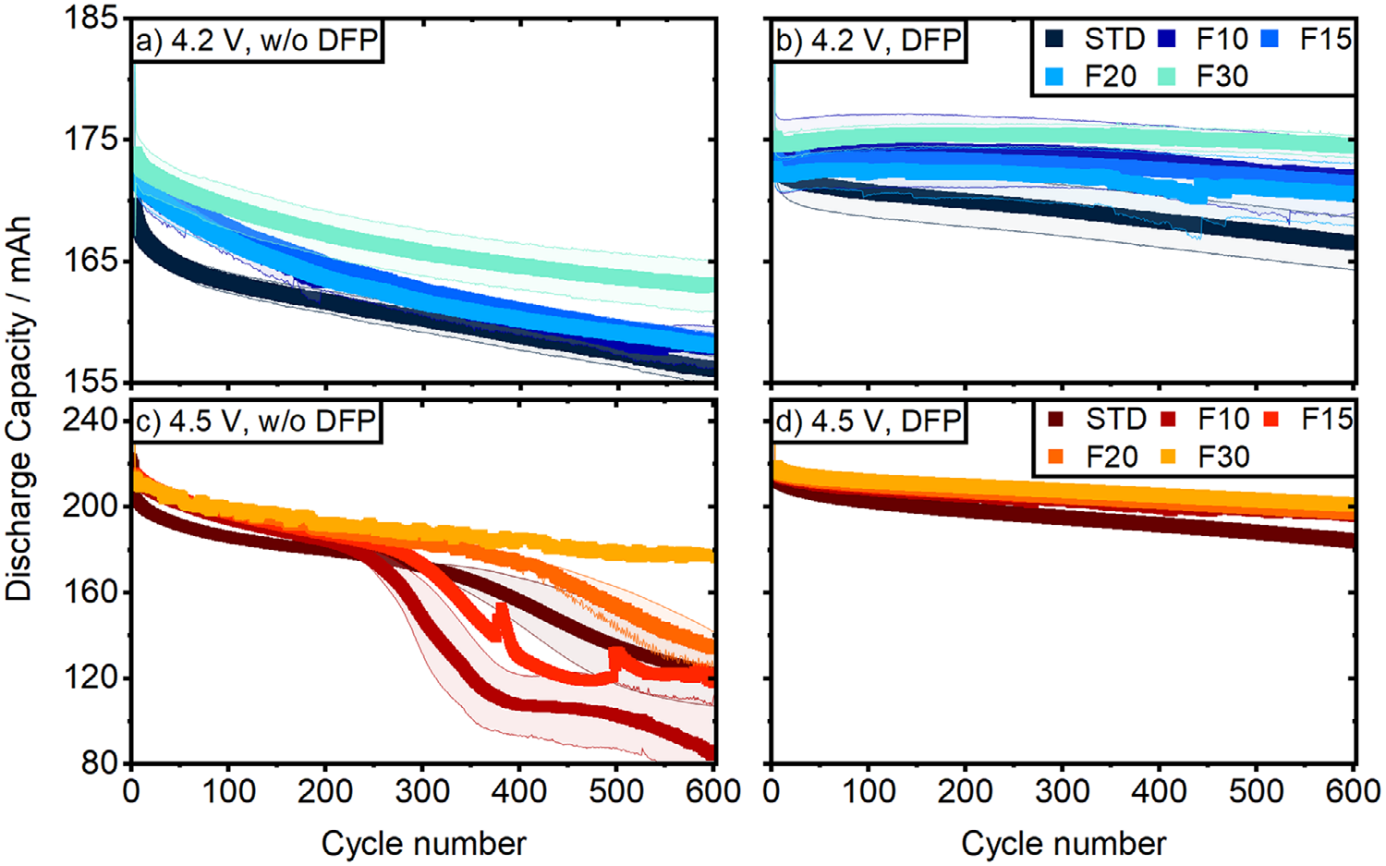
Capacity development of pouch cells (NMC622||AG, 200 mAh, 1.0 C) for 600 cycles and 4.2 V (a, b) and 4.5 V (c, d) UCVs. Different electrolytes with increasing FEC as a co‐solvent were employed, depicted with different colors. In addition, the additive DFP (b and d) resulted in increased capacity retention.

### 3.2 Surface Analysis

The main advantage of DFP as an electrolyte additive is preventing transition metal deposition on the negative electrode. With DFP and FEC in the same electrolyte, an even further reduction of transition metal deposition is suggested. The amount of transition metal deposited on the negative electrode surface is shown in Figure 2.

**FIGURE 2:**
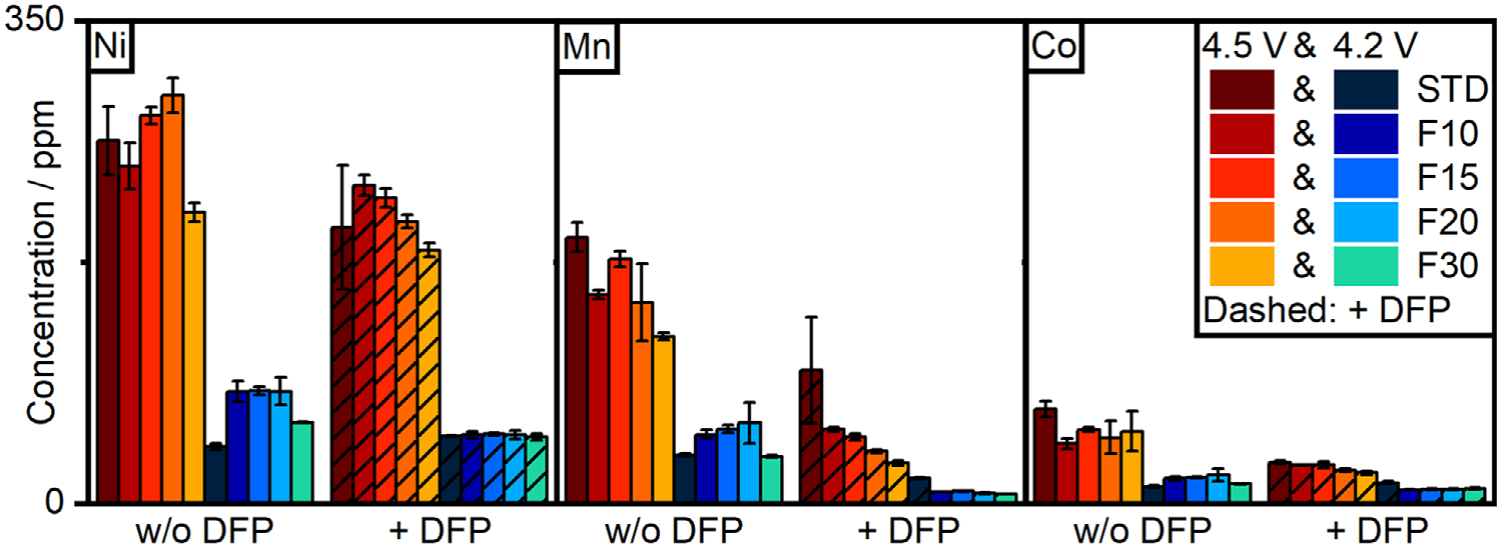
Concentration of deposited transition metals on the negative electrode surface (graphite) transported from the positive electrode (NMC622). Both 4.5 and 4.2 V UCVs and all electrolytes, with and without the addition of DFP, were investigated. The red gradient represents 4.5 V UCV, the blue gradient 4.2 V UCV, and the dashed bars the addition of DFP. The quantification of Ni, Co, and Mn was performed via IPC‐OES.

The highest amount of transition metals was found for Ni, followed by Mn, with the lowest amount for Co matching literature data for the employment of NMC622 and increased UCVs [38]. At higher voltages of 4.5 V, Ni, which constitutes the highest proportion of transition metals in the NMC622 cathode, is most frequently deposited on the negative electrode. For all electrolytes without DFP, no trend is observed, with only F30 showing decreased amounts, which suggests that the earlier start of the rollover effect is promoted more by continuous electrolyte decomposition than by transition metal deposition, as the measurements were all performed after 600 cycles. Adding DFP reduces the amount of Ni only marginally, but a decrease can be seen within the electrolytes from F10 to F30, matching electrochemical data. For 4.2 V UCV, the Ni deposition is similar for all electrolytes with a slight reduction for DFP electrolytes. Lower UCVs cause less degradation of the cathode material, ultimately leading to reduced transition metal deposition [17, 39]. Despite lower amounts of Mn within the electrode, increased concentrations are observed. For 4.5 V UCV and increasing amounts of FEC within the electrolyte, lower deposited Mn is found. The addition of DFP reduces the deposition even further. At lower UCVs, a general reduction is observed, and for DFP nearly no deposition is found. Co‐deposition on the negative electrode was minimal for all electrolytes, with an overall decrease observed both for 4.2 V UCV and upon DFP addition. Given the generally minor changes in all three transition metals deposited on the negative electrode, the suppression of the rollover effect upon DFP addition is driven by metal complexation and, at the same time, by reduced electrolyte reactivity, which is discussed in chapter 3.3. SEM images of the negative electrodes of STD, F15, and F30 (with and without DFP, Figure S6) for UCV 4.5 V were taken. The images revealed homogeneous surface morphologies for each sample without pronounced localized cracking or deposits. Therefore, the surface measurements can be considered representative, confirming the reliability of the *post‐mortem* analysis.

Further negative electrode surface measurements were performed with XPS of anodes for STD, F15, and F30 (with and without DFP) and increased UCV (Figure 3).

**FIGURE 3:**
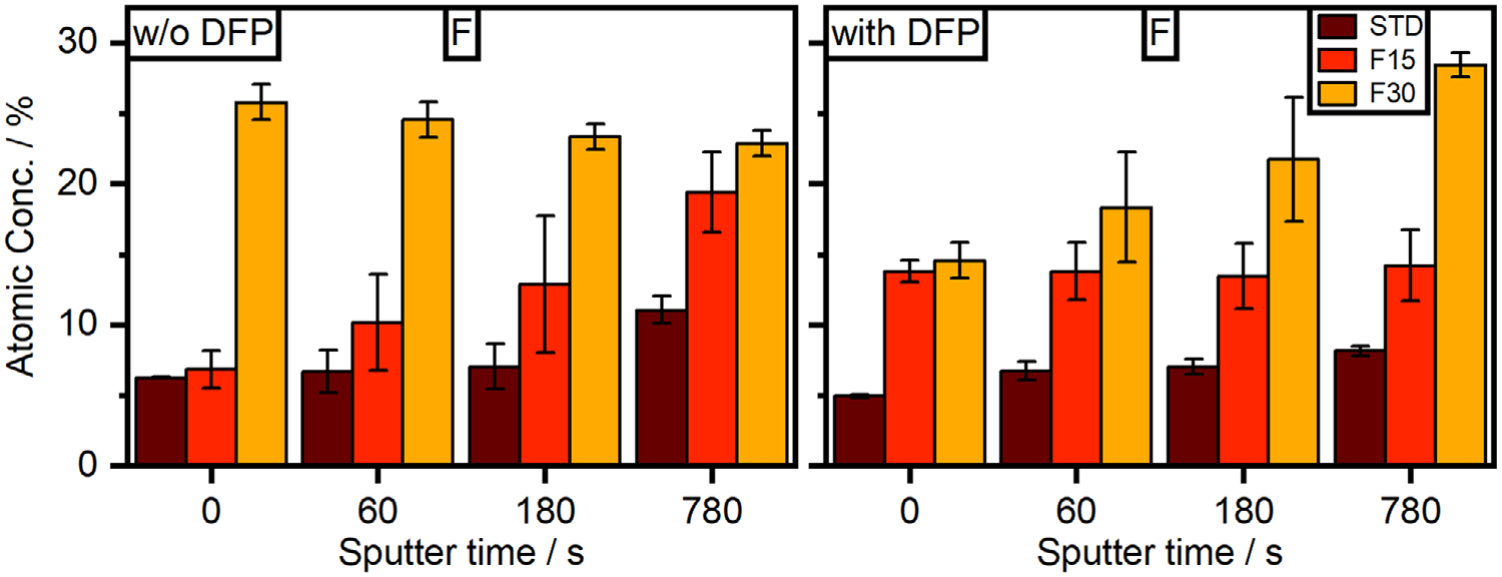
Atomic concentration of F based on F 1s measured with XPS depth profiling of anode surfaces of STD, F15 and F30 for UCV 4.5 V with and without the addition of DFP.

XPS depth profiling of the anode surfaces (UCV 4.5 V) showed an increasing F atomic concentration with increasing sputtering time and increasing FEC content in the electrolyte. Consistent F atomic concentration in F30 without DFP and F15 with DFP led to the assumption that the organic top layer of the SEI was damaged during sample preparation. This suggests enhanced formation of fluorine‐containing SEI components. The distribution of fluorinated compounds within the SEI is non‐uniform and suggests the formation of inorganic fluoride species, particularly LiF, closer to the bulk. LiF is primarily formed by abstraction from FEC, matching increased concentration with higher FEC content. The addition of DFP does not influence the F 1s atomic concentration considerably, which does not correlate with the electrochemical data, where the rollover effect was observed for STD and F15 electrolytes. However, a general increase of LiF content in the SEI is known to be beneficial for battery performance [40].

### 3.3 Electrolyte Investigations

The synergistic effect of the addition of DFP, high FEC, and thus low EC solvent content can be attributed to the prevention of the formation of FEC‐ and EC‐containing degradation products. HPLC measurements of electrolytes can detect degradation products soluble in the electrolyte, thus giving insightinto the electrolyte's state of health. As only soluble analytes are detectable, only assumptions of the composition of the SEI can be stated. Electrolyte components tend to polymerize during cell operation, which allows shorter oligomers that are still soluble to be detected. Consequently, aging mechanisms and SEI components can be reliably predicted, as already shown in studies by Henschel et al. [30]. General comparison of the electrolyte degradation is possible via total ion chromatograms (TIC) of the extracted electrolytes (Figure 4).

**FIGURE 4:**
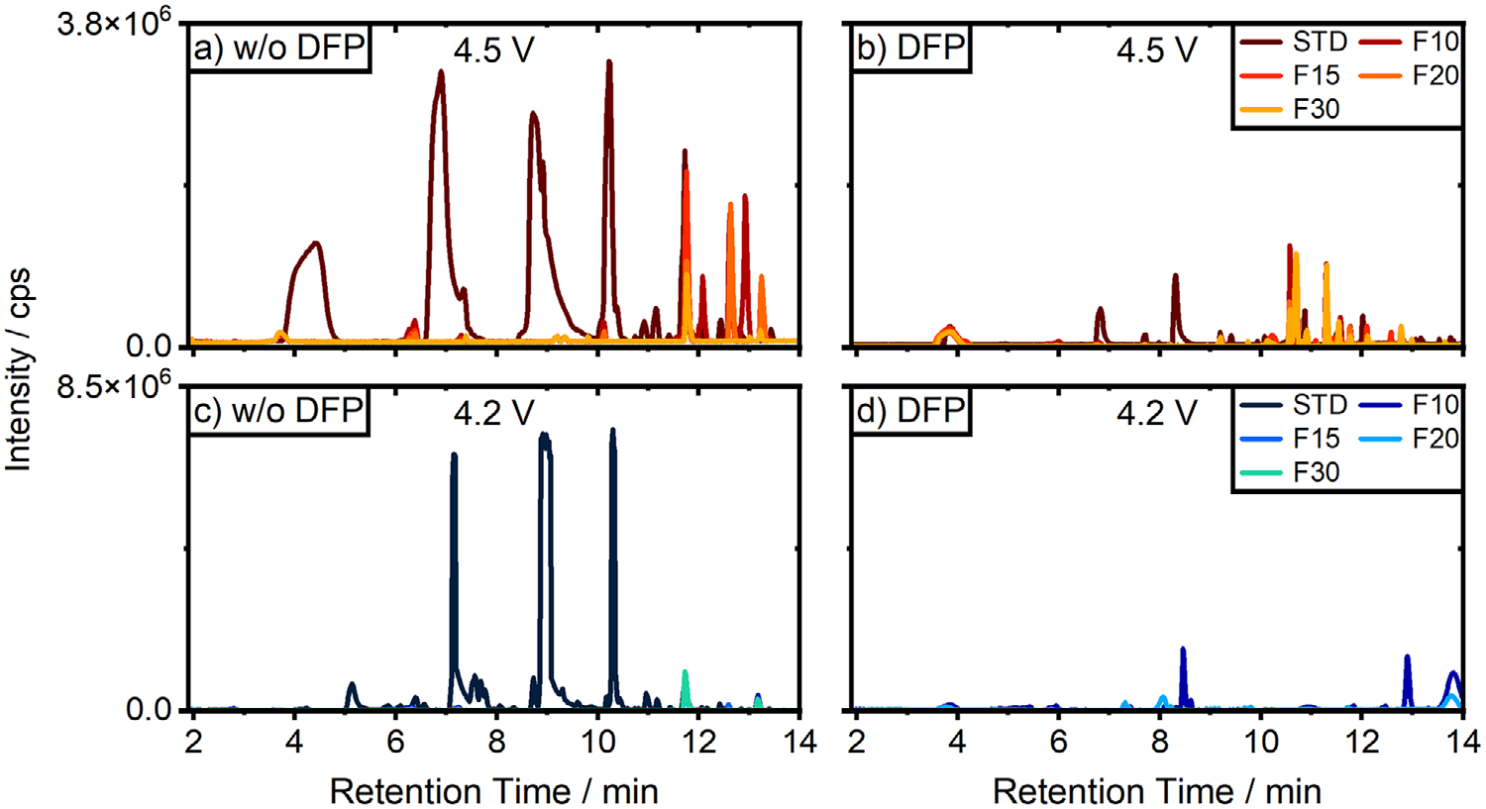
TICs for HPLC‐QToF measurements of electrochemically active electrolytes with an increasing amount of FEC and with (b and d) or without (a and c) the use of DFP. Additionally, the electrolytes were investigated in terms of their aging for different 4.2 V UCV (blue gradient) and 4.5 V UCV (red gradient).

For electrolytes without DFP, the most intense peaks are observable for STD (Figure 4a,c) for both UCVs. All visible peaks represent EC and EMC degradation products and are thus reduced for all FEC‐containing electrolytes. Typical degradation products of EC and EMC are carbonate oligomers or carbonate glycol co‐oligomers, which have been reported by Henschel et al. [41]. Smaller peaks visible in the TIC of F10‐F30 are related to FEC degradation products but with reduced intensity compared to STD electrolytes. Thus, electrolyte degradation is reduced with the use of FEC, indicating a better‐formed SEI and less EC and EMC decomposition, which is explained in detail in the following chapter, 3.4. Overall, the use of FEC results in an increased state of health of the electrolyte. The additive DFP is known in the literature for its effect in preventing transition metal dissolution for use in high‐voltage applications [42, 43]. The effect prevents the deposition of transition metals on the anode surface, ultimately leading to less lithium plating and electrolyte degradation. In addition to this effect, for the electrolytes with DFP, not only are the degradation products originating from EC and EMC reduced, but also the FEC and EC/EMC related degradation products exhibit lower signal intensities. For 4.5 V UCV (Figure 4b), the decrease in intensity matches the electrochemical data compared to the electrolytes without DFP. Thus, the addition of DFP not only hinders the rollover effect for 4.5 V UCV but also reduces electrolyte degradation by improving the electrolyte's state of health. The highest reduction in TIC intensities is observed for the 4.2 V UCV and the addition of DFP. After 600 no intense peaks can be observed in the TICs, confirming the retained state of health of the electrolyte. Ultimately, the combination of FEC and DFP in the electrolyte further improves the state of health of the electrolyte, enabling better long‐term stability matching cycling data for both 4.2 and 4.5 V UCVs. The reduced electrolyte degradation is also visible in the overall increased capacity for 600 cycles, and all investigated electrolytes with the additive DFP.

### 3.4 Influence of FEC and DFP Additive on Electrolyte Degradation

The TICs alone are not indicative of the number and quantity of degradation products. The peaks of low‐concentration molecules are not intense enough to be visible in the TIC of HPLC‐QToF measurements. The amount or variety of degradation products can vary despite low intensities in the TIC, as the ionization of said products highly depends on the structure of the molecule and its ability to be ionized. Thus, the peak areas of one molecule can be compared semi‐qualitatively but do not allow the quantification of the exact amount in the electrolyte. Different peak areas of exemplary selected degradation products are presented in Figure 5. These include FEC‐based species (a and b), carbonate‐based species (c), and phosphate‐based species (d).

**FIGURE 5:**
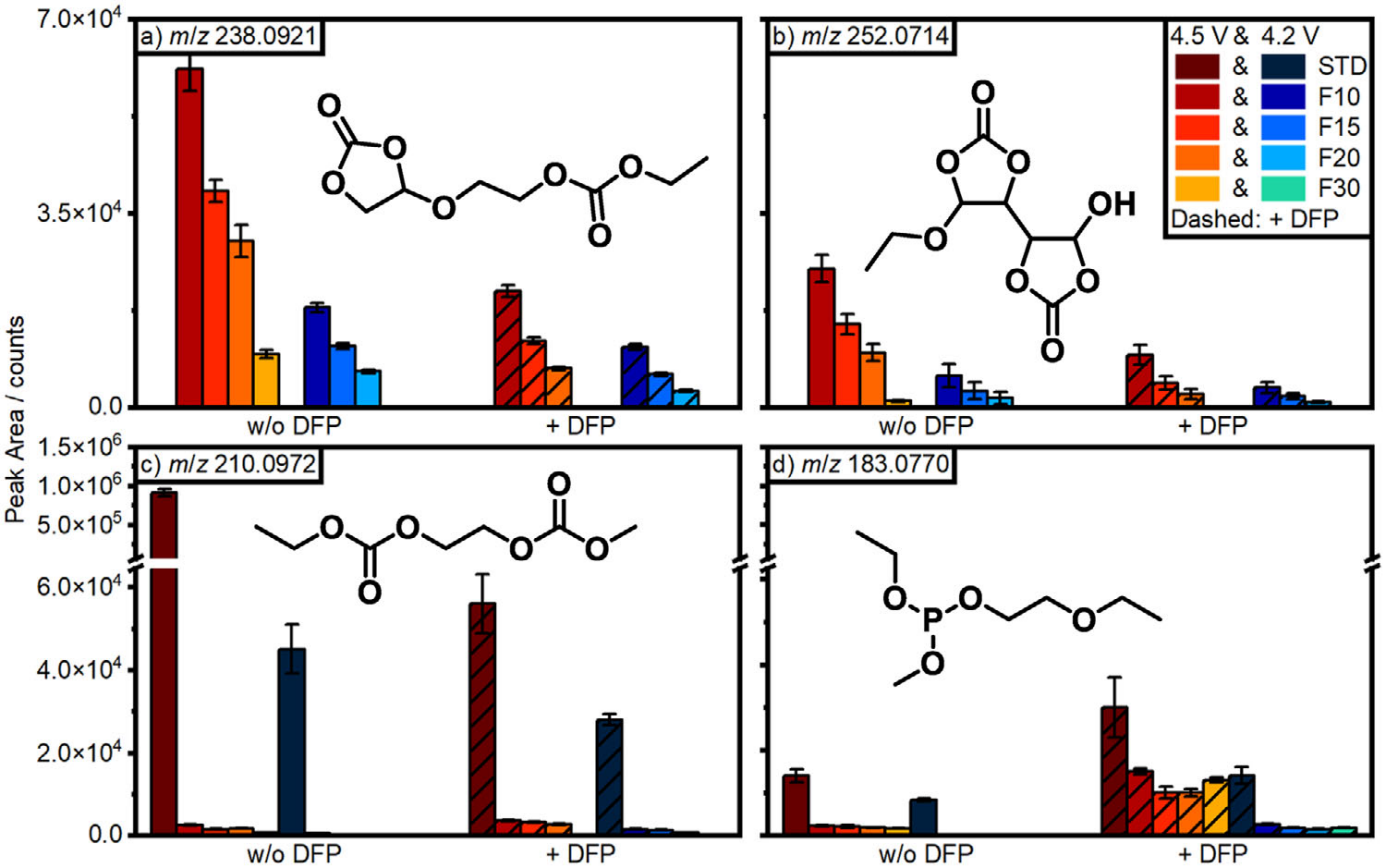
Peak areas of different EICs for degradation products detected via HPLC‐QToF. FEC‐related products (a and b), carbonate‐based and phosphate‐based products (c and d) are shown for all investigated electrolytes. Both investigated 4.5 and 4.2 V UCVs are shown with a red color gradient and a blue color gradient, respectively.

For both FEC‐based degradation products (Figure 5a,b), no peak areas are observed in STD electrolytes for both UCVs, as no in situ VC is formed in those electrolytes. For the FEC‐containing electrolytes, the peak areas decrease with increasing FEC content, also matching literature, as the in situ formed VC reacts with EC‐based degradation products, forming displayed products [24, 44]. High‐voltage applications tend to increase electrolyte degradation, represented by the generally increased peak areas of 4.5 V compared to the 4.2 V application. Additionally, strongly decreased peak areas of displayed FEC‐based degradation products (Figure 5a,b) are observed for F30 due to the absence of EC, which can also initiate the formation of observed degradation reactions. Peak areas of one typical carbonate‐based degradation product (dimethyl‐2,5‐dioxahexane dicarboxylate (DMDOHC)) are displayed in Figure 5c. The highest peak areas are observed for the STD and STD+DFP electrolytes for both UCVs, as no FEC is employed, which would reduce the electrolyte reactivity and the amount of EC itself. The ratio of the peak areas of STD for 4.5 V and STD for 4.2 V corresponds to a reduction of 95.1%, whereby the ratio is reduced to 50.0% for the addition of DFP. Comparing the peak areas for STD and STD+DFP at 4.5 V UCV, a reduction of 93.8% is achieved, and 37.8% for 4.2 V, matching the already known assumption of decreased electrolyte degradation for DFP employment. Electrolytes containing FEC only show peak areas for 4.5 V and not 4.2 V UCV, as FEC decreases overall electrolyte reactivity. The peak areas of another typical degradation product, which is phosphate‐glycol based, are shown in Figure 5d. The degradation product has only been detected in the STD electrolyte without DFP and with 4.2 V UCV, while increased peak areas have been observed in all electrolytes with 4.5 V UCV. For the addition of DFP, an overall increase in intensity is observable, as DFP also decomposes, forming similar or the same degradation products. With the addition of FEC in the electrolyte, the overall peak areas for phosphate‐based products can be reduced according to the assumption that FEC decreases electrolyte reactivity. The general trend of peak areas for both UCVs also matches the trend for previously shown degradation products, where the increased UCV induces more electrolyte aging. The reduced peak areas of the displayed degradation products may point to a degradation reaction between FEC and DFP, potentially producing organofluorophosphate‐containing polyVC structures, consistent with the proposal by Kubota et al. [43]. Electrochemical performance is best when using a combination of FEC and DFP, which could be explained by a modified SEI/CEI and thus prevention of the rollover effect, consistent with general electrolyte degradation and long‐term stability.

### 3.5 Structure Elucidation of FEC and DFP‐Based Degradation Products

Phosphate‐based molecules have been shown to counteract the rollover effect in high voltage applications [14, 45, 46]. The addition of DFP results in higher concentrations of degradation products originating from PF_6–_, as DFP is already a degradation product of PF_6–_ itself [47, 48]. Thus, the reaction between in situ VC and DFP degradation products is plausible and can be verified via HPLC‐QToF. For structure elucidation, the fragmentation spectrum of a chosen FEC and DFP‐based degradation product is displayed in Figure 6.

**FIGURE 6:**
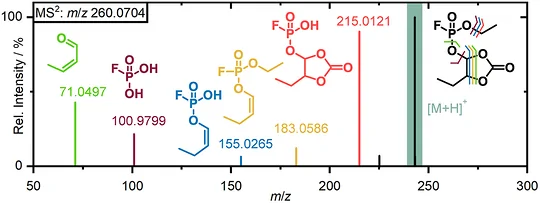
MS^2^ spectra of a FEC degradation product with *m*/*z* 260.0704 [M+NH_4_]^+^. The fragmentation experiments were conducted for structure elucidation. MS^2^ fragments are assigned to their respective formed structure and masses.

The precursor ion with *m*/*z* 260.0704 [M+NH_4_]^+^ was chosen for fragmentation experiments. The postulated sum formula C_7_H_12_O_6_FP has a relative mass deviation of <5 ppm (*m*/*z*_calc._ = 260.0694 [M+NH_4_]^+^) and a double bond equivalent (DBE) of 2. MS^2^ spectrum (Figure 6) indicates the presence of a terminal C_2_H_4_ bond to the phosphate backbone, as the fragment *m*/*z* 215.0121 [M+H]^+^ correlates to C_5_H_8_O_6_FP. The same loss of C_2_H_4_ is observed for fragments *m*/*z* 155.0265 [M+H]^+^ and *m*/*z* 100.9799 [M+H]^+^, correlating to the sum formulas C_4_H_8_O_3_FP and H_2_O_3_FP, respectively. The fragment *m*/*z* 100.9799 reveals the molecule originating from DFP/PF_6_^−^ degradation, as both end groups are subtracted and only the OFP backbone remains. Additionally, the fragment *m*/*z* 155.0265 [M+H]^+^ not only correlates to the loss of C_2_H_4_ but also to a loss of CO_3_, revealing the presence of a VC unit in the molecule. The alkyl chain remains bound to the OFP backbone and contains a double bond at the position of the VC moiety. The presence of VC within the molecule is further supported by fragment *m*/*z *183.0586 [M+H]^+^, corresponding to a sum formula of C_4_H_8_O_3_FP. The mass difference between the parent ion and the fragment again correlates to the loss of CO_3_, which further supports the assumption of a VC unit within the molecule. The fragment *m*/*z* 71.0497 [M+H]^+^ correlates to the subtraction of OFP backbone and VC, leaving only the terminal alkyl chain and a terminal aldehyde corresponding to the sum formula C_4_H_6_O. All MS^2^ fragments support the formation of FEC‐ and DFP‐based degradation products, matching previously postulated literature by Kubot et al. [43]. They also postulated a positive effect of FEC and DFP in the range of additive concentrations in the same electrolyte regarding their toxicity and performance. Thus, the reaction between DFP and FEC appears advantageous for battery performance. Only polyVC products were found within the electrolyte, and no degradation products which follow a ring‐opening reaction. The degradation mechanism between FEC and DFP can therefore be described as a combination of both individual mechanisms and is displayed in Figure 7 [30, 42, 44].

**FIGURE 7:**
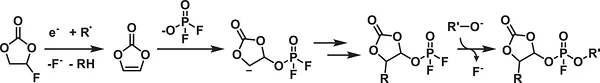
Postulated reaction scheme of the degradation reaction between FEC and DFP based on already postulated mechanisms [30, 42, 44]. The reaction follows the initial in situ formation of VC followed by a nucleophilic attack of the DFP, with subsequent reactions forming the found degradation products.

The reaction between FEC and DFP can be described first with an e^−^ transfer to FEC followed by a subtraction of F^−^. Afterwards, in situ VC is formed via an additional hydrogen subtraction. The in situ VC has been shown to react with carbonate‐based degradation products to form carbonate and VC co‐oligomers. With the addition of DFP, the more favorable reaction can be assumed to follow a nucleophilic polymerization reaction. The negatively charged oxygen of the DFP initiated the chain reaction by attacking the double bond of the VC. Subsequent reactions can then substitute fluoride with carbonate or glycol‐based degradation products, forming found degradation products. The performance‐enhancing properties of FEC‐based electrolytes with the additive DFP can therefore be attributed to this reaction, ultimately enhancing SEI performance. Simultaneously, conventional aging mechanisms are also suppressed, which contributes positively to improving long‐term cycling compared to STD+DFP electrolytes.

## 4 Conclusion

In this study, electrolyte investigations in cells for high‐voltage applications were carried out. The electrolytes contained varying contents of FEC and EC, including the replacement of EC by FEC and the addition of the electrolyte additive DFP. Long‐term cycling experiments for 600 cycles with 4.5 and 4.2 V upper cut‐off voltages (UCV) were applied. At 4.2 V UCV, no rollover effect was observed, with the best performance at high FEC contents, where FEC replaced EC. The addition of DFP increased the capacity of all electrolytes; again, FEC replacing EC showed the best long‐term stability. At 4.5 V UCV, the use of FEC and EC as cosolvents without DFP revealed severe aging and poor electrochemical performance worse than STD. With the addition of DFP, the rollover effect is negated, resulting in similar trends that increased FEC content results in improved electrochemical stability.

Electrolyte investigations by means of HPLC‐QToF were carried out to investigate the electrolyte's state of health for the addition of DFP. STD electrolytes exhibited the largest intensities of the corresponding degradation products. Utilizing FEC electrolytes decreased the amount of degradation, with F30 (1 M LiPF_6_ FEC:EMC 3:7) showing the least degradation products at both UCV. The addition of DFP revealed decreased electrolyte degradation compared to electrolytes without DFP, despite following the same trend with lower amounts of degradation products for higher FEC contents. Additionally, ICP‐OES measurements revealed that transition metal deposition is increased for 4.5 V with a slight reduction for the employment of DFP in all electrolytes. Overall, the reaction of FEC and DFP could be attributed to improved electrochemical performance, particularly a better state of health of the electrolyte at higher cycle numbers. This study showed that the use of FEC and DFP for high‐voltage applications leads to improved long‐term stability and enables the use of EC and FEC as co‐solvents by counteracting their detrimental effects.

## Conflicts of Interest

The authors declare no conflicts of interest.

## Supporting information




**Supporting File**: smtd70857‐sup‐0001‐SuppMat.docx.

## Data Availability

The data that support the findings of this study are available from the corresponding author upon reasonable request.

## References

[1] C. P. Grey and D. S. Hal , “Prospects for Lithium‐Ion Batteries and Beyond—A 2030 Vision,” Nature Communications 11 (2020): 6279, 10.1038/s41467-020-19991-4. PMC7722877 33293543

[2] K. Xu , “Electrolytes and Interphases in Li‐Ion Batteries and Beyond,” Chemical Reviews 114 (2014): 11503–11618, 10.1021/cr500003w. 25351820

[3] K. Xu , "Electrolytes, Interfaces and Interphases," *Royal Society Of Chemistry*, (2023), London.

[4] E. Peled and S. Menki , “Review—SEI: Past, Present and Future,” Journal of the Electrochemical Society 164 (2017): A1703–A1719, 10.1149/2.1441707jes.

[5] L. Wang , A. Menakath , F. Han , et al., “Identifying the Components of the Solid–Electrolyte Interphase in Li‐Ion Batteries,” Nature Chemistry 11 (2019): 789–796, 10.1038/s41557-019-0304-z. 31427766

[6] J. Vetter , P. Novák , M. R. Wagner , et al., “Ageing Mechanisms in Lithium‐Ion Batteries,” Journal of Power Sources 147 (2005): 269–281, 10.1016/j.jpowsour.2005.01.006.

[7] T. Joshi , K. Eom , G. Yushin , and T. F. Fulle , “Effects of Dissolved Transition Metals on the Electrochemical Performance and SEI Growth in Lithium‐Ion Batteries,” Journal of The Electrochemical Society 161 (2014): A1915–A1921, 10.1149/2.0861412jes.

[8] Y. Song , L. Wang , L. Sheng , et al., “The Significance of Mitigating Crosstalk in Lithium‐Ion Batteries: A Review,” Energy & Environmental Science 16 (2023): 1943–1963, 10.1039/D3EE00441D.

[9] E. Björklund , C. Xu , W. M. Dose , et al., “Cycle‐Induced Interfacial Degradation and Transition‐Metal Cross‐Over in LiNi_0.8_Mn_0.1_Co_0.1_O_2_–Graphite Cells,” Chemistry of Materials 34 (2022): 2034–2048, 10.1021/acs.chemmater.1c02722. PMC9082506 35557994

[10] P. M. Attia , A. Bills , F. Brosa Planella , et al., “Review—“Knees” in Lithium‐Ion Battery Aging Trajectories,” Journal of The Electrochemical Society 169 (2022): 060517, 10.1149/1945-7111/ac6d13.

[11] D. Aurbac , “Electrode–Solution Interactions in Li‐Ion Batteries: A Short Summary and New Insights,” Journal of Power Sources 119–121 (2003): 497–503, 10.1016/S0378-7753(03)00273-8.

[12] J. A. Gilbert , I. A. Shkrob , and D. P. Abraha , “Transition Metal Dissolution, Ion Migration, Electrocatalytic Reduction and Capacity Loss in Lithium‐Ion Full Cells,” Journal of The Electrochemical Society 164 (2017): A389–A399, 10.1149/2.1111702jes.

[13] H. Zhao , X. Yu , J. Li , et al., “Film‐Forming Electrolyte Additives for Rechargeable Lithium‐Ion Batteries: Progress and Outlook,” Journal of Materials Chemistry A 7 (2019): 8700–8722, 10.1039/C9TA00126C.

[14] X. Fan and C. Wan , “High‐Voltage Liquid Electrolytes for Li Batteries: Progress and Perspectives,” Chemical Society Reviews 50 (2021): 10486–10566, 10.1039/D1CS00450F. 34341815

[15] A. M. Haregewoin , A. S. Wotango , and B. J. Hwan , “Electrolyte Additives for Lithium Ion Battery Electrodes: Progress and Perspectives,” Energy & Environmental Science 9 (2016): 1955–1988, 10.1039/C6EE00123H.

[16] L. Dong , S. Zhong , B. Yuan , et al., “Electrolyte Engineering for High‐Voltage Lithium Metal Batteries,” Research 2022 (2022): 9837586, 10.34133/2022/9837586. PMC9470208 36128181

[17] S. Klein , L. Haneke , P. Harte , et al., “Suppressing Electrode Crosstalk and Prolonging Cycle Life in High‐Voltage Li Ion Batteries: Pivotal Role of Fluorophosphates in Electrolytes,” ChemElectroChem 9 (2022): 202200469, 10.1002/celc.202200469.

[18] R. Li , P. Zhang , J. Huang , et al., “Enhanced High Voltage Performance of LiNi_0.5_Mn_0.3_Co_0.2_O_2_ Cathode via the Synergistic Effect of LiPO_2_F_2_ and FEC in Fluorinated Electrolyte for Lithium‐Ion Batteries,” RSC Advances 11 (2021): 7886–7895, 10.1039/D0RA10280F. PMC8695088 35423334

[19] A. Wang , L. Wang , H. Liang , et al., “Lithium Difluorophosphate as a Widely Applicable Additive to Boost Lithium‐Ion Batteries: A Perspective,” Advanced Functional Materials 33 (2023): 2211958, 10.1002/adfm.202211958.

[20] S. Klein , P. Harte , S. Van Wickeren , et al., “Re‐Evaluating Common Electrolyte Additives for High‐Voltage Lithium Ion Batteries,” Cell Reports Physical Science 2 (2021): 100521, 10.1016/j.xcrp.2021.100521.

[21] L. Ma , L. Ellis , S. L. Glazier , X. Ma , and J. R. Dah , “Combinations of LiPO_2_F_2_ and Other Electrolyte Additives in Li[Ni_0.5_Mn_0.3_Co_0.2_]O_2_/Graphite Pouch Cells,” Journal of The Electrochemical Society 165 (2018): A1718–A1724, 10.1149/2.0661809jes.

[22] L. Hu , Z. Zhang , and K. Amin , “Fluorinated Electrolytes for Li‐Ion Battery: An FEC‐Based Electrolyte for High Voltage LiNi_0.5_Mn_1.5_O_4_/Graphite Couple,” Electrochemistry Communications 35 (2013): 76–79, 10.1016/j.elecom.2013.08.009.

[23] J. Tan , J. Matz , P. Dong , J. Shen , and M. Y , “A Growing Appreciation for the Role of LiF in the Solid Electrolyte Interphase,” Advanced Energy Materials 11 (2021): 2100046, 10.1002/aenm.202100046.

[24] N. Fehlings , M. Weiling , J. Hesper , et al., “Investigation of Degradation Pathways in Fluoroethylene Carbonate Based Electrolytes via Chromatographic Techniques,” Batteries & Supercaps 9 (2025): 202500610, 10.1002/batt.202500610.

[25] B. Wang , H. A. Doan , S. B. Son , et al., “Data‐Driven Design of Electrolyte Additives Supporting High‐Performance 5 V LiNi_0.5_Mn_1.5_O_4_ Positive Electrodes,” Nature Communications 16 (2025): 3413, 10.1038/s41467-025-57961-w. PMC11986164 40210879

[26] K. Duan , J. Ning , L. Zhou , et al., “Synergistic Inorganic–Organic Dual‐Additive Electrolytes Enable Practical High‐Voltage Lithium‐Ion Batteries,” ACS Applied Materials & Interfaces 14 (2022): 10447–10456, 10.1021/acsami.1c24808. 35179877

[27] X. Zheng , G. Fang , Y. Pan , Q. Li , and M. W , “Synergistic Effect of Fluoroethylene Carbonate and Lithium Difluorophosphate on Electrochemical Performance of SiC‐Based Lithium‐Ion Battery,” Journal of Power Sources 439 (2019): 227081, 10.1016/j.jpowsour.2019.227081.

[28] Y. P. Stenzel , F. Horsthemke , M. Winter , and S. Nowa , “Chromatographic Techniques in the Research Area of Lithium Ion Batteries: Current State‐of‐the‐Art,” Separations 6 (2019): 26, 10.3390/separations6020026.

[29] C. Peschel , F. Horsthemke , M. Leißing , et al., “Analysis of Carbonate Decomposition During Solid Electrolyte Interphase Formation in Isotope‐Labeled Lithium Ion Battery Electrolytes: Extending the Knowledge About Electrolyte Soluble Species,” Batteries & Supercaps 3 (2020): 1183–1192, 10.1002/batt.202000170.

[30] J. Henschel , C. Peschel , S. Klein , F. Horsthemke , M. Winter , and S. Nowa , “Clarification of Decomposition Pathways in a State‐of‐the‐Art Lithium Ion Battery Electrolyte Through 13 C‐Labeling of Electrolyte Components,” Angewandte Chemie International Edition 59 (2020): 6128–6137, 10.1002/anie.202000727. PMC7187180 32012404

[31] M. Evertz , J. Kasnatscheew , M. Winter , and S. Nowa , “Investigation of Various Layered Lithium Ion Battery Cathode Materials by Plasma‐ and X‐ray‐Based Element Analytical Techniques,” Analytical and Bioanalytical Chemistry 411 (2019): 277–285, 10.1007/s00216-018-1441-8. 30374724

[32] S. Wilschefski and M. Baxte , “Inductively Coupled Plasma Mass Spectrometry: Introduction to Analytical Aspects,” Clinical Biochemist Reviews 40 (2019): 115–133, 10.33176/AACB-19-00024. PMC6719745 31530963

[33] J. Henschel , J. M. Dressler , M. Winter , and S. Nowa , “Reaction Product Analyses of the Most Active “Inactive” Material in Lithium‐Ion Batteries—The Electrolyte. I: Themal Stress and Marker Molecules,” Chemistry of Materials 31 (2019): 9970–9976, 10.1021/acs.chemmater.9b04133.

[34] R. Schmid , S. Heuckeroth , A. Korf , et al., “Integrative Analysis of Multimodal Mass Spectrometry Data in MZmine 3,” Nature Biotechnology 41 (2023): 447–449, 10.1038/s41587-023-01690-2. PMC10496610 36859716

[35] B. Vortmann‐Westhoven , M. Winter , and S. Nowa , “Where Is the Lithium? Quantitative Determination of the Lithium Distribution in Lithium Ion Battery Cells: Investigations on the Influence of the Temperature, the C‐Rate and the Cell Type,” Journal of Power Sources 346 (2017): 63–70, 10.1016/j.jpowsour.2017.02.028.

[36] I. Buchberger , S. Seidlmayer , A. Pokharel , et al., “Aging Analysis of Graphite/LiNi_1/3_Mn_1/3_Co_1/3_O_2_ Cells Using XRD, PGAA, and AC Impedance,” Journal of The Electrochemical Society 162 (2015): A2737–A2746, 10.1149/2.0721514jes.

[37] S. Klein , P. Bärmann , L. Stolz , et al., “Demonstrating Apparently Inconspicuous but Sensitive Impacts on the Rollover Failure of Lithium‐Ion Batteries at a High Voltage,” ACS Applied Materials & Interfaces 13 (2021): 57241–57251, 10.1021/acsami.1c17408. 34813694

[38] S. J. Wachs , C. Behling , J. Ranninger , J. Möller , K. J. J. Mayrhofer , and B. B. Berke , “Online Monitoring of Transition‐Metal Dissolution From a High‐Ni‐Content Cathode Material,” ACS Applied Materials & Interfaces 13 (2021): 33075–33082, 10.1021/acsami.1c07932. 34232020

[39] J. Li , L. E. Downie , L. Ma , W. Qiu , and J. R. Dah , “Study of the Failure Mechanisms of LiNi_0.8_Mn_0.1_Co_0.1_O_2_ Cathode Material for Lithium Ion Batteries,” Journal of The Electrochemical Society 162 (2015): A1401–A1408, 10.1149/2.1011507jes.

[40] X. Fan , X. Ji , F. Han , et al., “Fluorinated Solid Electrolyte Interphase Enables Highly Reversible Solid‐State Li Metal Battery,” Science Advances 4 (2018): aau9245, 10.1126/sciadv.aau9245. PMC6303121 30588493

[41] J. Henschel , C. Peschel , F. Günter , G. Reinhart , M. Winter , and S. Nowa , “Reaction Product Analysis of the Most Active “Inactive” Material in Lithium‐Ion Batteries—The Electrolyte. II: Battery Operation and Additive Impact,” Chemistry of Materials 31 (2019): 9977–9983, 10.1021/acs.chemmater.9b04135.

[42] C. Wang , L. Yu , W. Fan , et al., “Lithium Difluorophosphate as a Promising Electrolyte Lithium Additive for High‐Voltage Lithium‐Ion Batteries,” ACS Applied Energy Materials 1 (2018): 2647–2656, 10.1021/acsaem.8b00342.

[43] M. Kubot , L. Frankenstein , E. Muschiol , et al., “Lithium Difluorophosphate: A Boon for High Voltage Li Ion Batteries and a Bane for High Thermal Stability/Low Toxicity: Towards Synergistic Dual Additives to Circumvent this Dilemma,” Chemsuschem 16 (2023): 202202189, 10.1002/cssc.202202189. 36533855

[44] Y. Jin , N.‐J. H. Kneusels , P. C. M. M. Magusin , et al., “Identifying the Structural Basis for the Increased Stability of the Solid Electrolyte Interphase Formed on Silicon With the Additive Fluoroethylene Carbonate,” Journal of the American Chemical Society 139 (2017): 14992–15004, 10.1021/jacs.7b06834. 28933161

[45] P. Shi , L. Zhang , H. Xiang , X. Liang , Y. Sun , and W. X , “Lithium Difluorophosphate as a Dendrite‐Suppressing Additive for Lithium Metal Batteries,” ACS Applied Materials & Interfaces 10 (2018): 22201–22209, 10.1021/acsami.8b05185. 29898366

[46] C.‐C. Su , M. He , C. Peebles , et al., “Functionality Selection Principle for High Voltage Lithium‐Ion Battery Electrolyte Additives,” ACS Applied Materials & Interfaces 9 (2017): 30686–30695, 10.1021/acsami.7b08953. 28820572

[47] C. Jayawardana , N. Rodrigo , and B. Parimalam , “Role of Electrolyte Oxidation and Difluorophosphoric Acid Generation in Crossover and Capacity Fade in Lithium Ion Batteries,” ACS Energy Letters 6 (2021): 3788–3792, 10.1021/acsenergylett.1c01657.

[48] S. Wiemers‐Meyer and M. Winter , “Mechanistic Insights Into Lithium Ion Battery Electrolyte Degradation—A Quantitative NMR Study,” Physical Chemistry Chemical Physics 18 (2016): 26595–26601, 10.1039/C6CP05276B. 27711648

