# Supplementary material for: Lithium Difluorophosphate and Fluoroethylene Carbonate Containing Electrolytes: How Instrumental Analytics Helps to Understand Synergistic Effects on Performance Improvement in High‐Voltage Li Battery Applications
Source: Small Methods. 2026 Jul 27;10(20):e70857. doi: 10.1002/smtd.70857 (PMC13706640; doi:10.1002/smtd.70857)
Supplement: Supplementary file 1 [file SMTD-10-e70857-s001.docx]

**Lithium Difluorophosphate and Fluoroethylene Carbonate Containing Electrolytes: How Instrumental Analytics Helps to Understand Synergistic Effects on Performance Improvement in High-Voltage Li Battery Applications**

Nick Fehlings^a^, Alexandros Tsoufios^a^, Tim Messink^a^, Martin Winter^a,b^, Simon Wiemers-Meyer^a^ and Sascha Nowak^a^*^*^*

*^a^ University of Münster, MEET Battery Research Center, Corrensstraße 46,
48149 Münster, Germany*

*^b^ Helmholtz-Institute Münster, IMD-4, FZ Jülich, Corrensstraße 46,
48149 Münster, Germany*

#### Supporting Information

Figure S 1: Charge-discharge curves (cycle 5, 50, 300 and 600) for UCV of 4.2 for electrolytes without DFP.

Figure S 2: Charge-discharge curves (cycle 5, 50, 300 and 600) for UCV of 4.2 for the electrolytes with DFP.

Figure S 3: Charge-discharge curves (cycle 5, 50, 300 and 600) for UCV of 4.5 for the electrolytes without DFP.

Figure S 4: Charge-discharge curves (cycle 5, 50, 300 and 600) for UCV of 4.5 for the electrolytes with DFP.

Figure S 5**:** Capacity Retention for both UCVs and electrolytes with and without the addition of DFP.

d)

e)

f)

c)

b)

a)

Figure S 6: Exemplarily shown surface SEM images of cycled anodes after 600 cycles for UCV of 4.5 V. a) STD, b) F15, c) F30, d) STD+DFP, e) F15+DFP, f) F30+DFP.

Figure S 7: Negative electrode F 1s core spectrum for the electrolytes STD, F15 and F30 with and without the addition of DFP. The peaks are labeled and their center is marked with a dotted line.
